# Femtosecond laser-assisted astigmatic keratotomy: a review

**DOI:** 10.1186/s40662-018-0099-9

**Published:** 2018-03-12

**Authors:** John S. M. Chang

**Affiliations:** Department of Ophthalmology, Hong Kong Sanatorium & Hospital, 8/F, Li Shu Pui Block, Phase II, 2 Village Road, Happy Valley, Hong Kong

**Keywords:** Femtosecond laser-assisted astigmatic keratotomy, Post-keratoplasty astigmatism, Astigmatism correction, Refractive surgery

## Abstract

**Background:**

Astigmatic keratotomy (AK) remains an accessible means to correct surgically induced or naturally occurring astigmatism. The advantages of femtosecond laser-assisted astigmatic keratotomy (FSAK) over conventional methods have been recognized recently.

**Main text:**

This review evaluates the efficacy, complications, and different methods of FSAK for correction of astigmatism in native eyes and those that underwent previous penetrating keratoplasty (PKP).

The penetrating and intrastromal FSAK (IFSAK) techniques can reduce post-keratoplasty astigmatism by 35.4% to 84.77% and 23.53% to 89.42%, respectively. In native eyes, the penetrating and IFSAK techniques reduce astigmatism by 26.8% to 58.62% and 36.3% to 58% respectively, implying that the magnitude of the astigmatic reduction is comparable between the two FSAK procedures. Nonetheless, IFSAK offers the additional advantages of almost no risk of infection, wound gape, and epithelial ingrowth. The use of nomograms, anterior-segment optical coherence tomography, and consideration of posterior cornea and corneal biomechanics are helpful to enhance the efficacy and safety of FSAK. The complications of FSAK in eyes that underwent PKP include overcorrection, visual loss, microperforations, infectious keratitis, allograft rejection, and endophthalmitis. The reported difficulties in native eyes include overcorrection, anterior gas breakthrough, and suction loss.

**Conclusions:**

In eyes that underwent PKP, FSAK effectively reduces high regular or irregular astigmatism, with rare and manageable complications. Nevertheless, the drawbacks of the procedure include the potential loss of visual acuity and low predictability. For native eyes undergoing femtosecond laser-assisted cataract surgery, IFSAK is a good choice to correct low astigmatism (< 1.5 diopters). The refractive effect of astigmatism from the posterior cornea needs to be considered in the nomograms for native eyes undergoing refractive cataract surgery. To further improve the efficacy of FSAK, more large-scale randomized studies with longer follow-up are needed.

## Background

Astigmatic keratotomy (AK), also known as arcuate keratotomy, has been performed for more than a century to correct astigmatism. With advances in technology, AK is performed with higher accuracy using a femtosecond laser compared with manual cutting with a blade.

Femtosecond laser-assisted cataract surgery is gaining popularity among surgeons, from 19% in 2014 to 29% in 2015 [1]. The femtosecond laser can be used not only to create capsulotomies and fragment the lens, but also to produce penetrating corneal or intrastromal incisions with high precision. Femtosecond laser-assisted AK (FSAK) is well proven to be effective and safe in reducing corneal astigmatism in highly astigmatic eyes after penetrating keratoplasty (PKP) [2, 3]. Patients who underwent PKP or deep anterior lamellar keratoplasty (DALK) might have substantial anisometropia; the primary goal of FSAK is the reduction of astigmatism after PKP to a level that allows the patient to wear contact lens or spectacles. This concept is important since the sequel of AK is somewhat unpredictable [4] and may require other visual aids. FSAK also can be performed to treat corneas that are too thin for refractive surgery or unsuitable for enhancement because of insufficient corneal tissue or severe dry eye [5].

## FSAK in post-keratoplasty eyes

### Summary of techniques

Three significant variables are present in AKs: optical zone diameter and the AK depth and arc length. The optical zone diameter usually is set at a fixed distance from 0.4 to 1 mm within the graft-host junction if no particular nomogram is used [6–12]. Regarding the depth, most AKs are penetrating, with the depth set at a fixed percentage of the thinnest pachymetry at the optical zone, ranging from 75% to 90%, or set based on the pre-existing corneal astigmatism [8, 11, 13]. Intrastromal AKs are performed 60 to 90 μm from the epithelium and 10% to 20% from the posterior cornea [5, 12, 14, 15]. The arc lengths of AK have been reported to range from 15 to 120 degrees. Most AKs are paired symmetrically along the steep axis. In some reports, single or asymmetric paired AKs were executed to correct irregular astigmatism [9, 11, 16]. The side-cut angles are mostly 90 degrees, except in the studies of Cleary et al. [11] and Rückl et al. [17] in which 135 degrees and 60 degrees were used, respectively.

### Efficacy

Among all reviewed studies, most eyes had undergone a PKP, and a small number had experienced DALK [7, 10, 13]/lamellar keratoplasty [8].

#### (i) FSAK vs. manual AK and mechanized AK

It has been suggested that the arc length, depth, and location precision can be better achieved in FSAK, compared with manual and mechanized AK [3, 18]. FSAK also is associated with lower risks of wound dehiscence, epithelial ingrowth, infection, and full-thickness corneal perforation [18].

Bahar et al. [19] reported a trend of better improvement in the uncorrected visual acuity (UCVA) and best-corrected visual acuity (BCVA) in the FSAK group compared with the manual AK group. However, the differences were not significant statistically (UCVA *p* = 0.2; BCVA *p* = 0.59), possibly due to the small sample size of 126. However, the improvements in the UCVA and BCVA were significant only in the FSAK group (Manual AK UCVA *p* = 0.09, BCVA *p* = 0.16; FSAK UCVA *p* = 0.004, BCVA *p* = 0.01). Also, the improvements in defocus equivalent and aberrations were slightly higher in the FSAK group (*p* = 0.31 and *p* = 0.65, respectively). One surgeon performed all AK procedures but used different techniques. The incisional depths differed between the groups, and the nomogram was modified in the last 10 FSAK subjects.

Hoffart et al. [18] compared the effectiveness between FSAK and mechanized AK performed by the same surgeon using the same nomogram. The changes in the mean UCVA (*p* = 0.735 and *p* = 0.194, respectively) and BCVA (*p* = 0.168 and *p* = 0.241, respectively) were not significant in the FSAK and mechanized AK groups. The refractive cylinder decreased more in the FSAK group (*p* = 0.011). Regarding the angle-of-error analysis, a less favorable outcome was observed in the mechanized AK group compared to the FSAK group (*p* = 0.052).

#### (ii) Penetrating FSAK

Penetrating FSAK involves cuts performed from the anterior surface. The wounds are closed, which decreases the incidence of wound infection. It is believed that the wound can be opened at a later follow-up examination if the effect of the astigmatic correction was insufficient. However, once the wound is opened, differential healing can cause significant overcorrection [16].

The preoperative keratometric astigmatic levels ranged from 4.4 diopters (D) [20] to 9.8 D [21], while postoperatively they ranged from 0.67 D to 5.2 D, respectively, in those studies. The keratometric astigmatic changes varied from 2.38 D [8] to 5.32 D [11] regardless of under- or overcorrection. The percentages of astigmatic reduction have ranged from 35.4% [8] to 84.77% [20]. The surgically induced astigmatism (SIA) has ranged from 1.577 D to 13.649 D [4]. A summary is shown in Table 1.

**Table 1:** Reviewed articles of FSAK performed in post-keratoplasty eyes

Reference	Type of FSAK	Open wound	Optical zone diameter	Incisional depth	Eyes (no.)	Eye type	Follow-up	Mean keratometric astigmatism (preoperative) (D)	Mean keratometric astigmatism (postoperative) (D)	% Reduction
St. Clair et al. (2016) [13]	Penetrating	Not reported	mean 6.66 mm (5.5–7.8 mm)	86% (range, 62%–94%)	89	Post-PKP/DALK	12 months	8.26 ± 2.90	3.62 ± 2.59	56.20%
Loriaut et al. (2015) [4]	Penetrating	✓	6–6.5 mm	75% of thinnest pachymetry	20	Post-PKP	17 ± 7.9 months	9.45 ± 2.97	4.64 ± 2.79	50.90%
Buzzonetti et al. (2009) [21]	Penetrating	✓	mean 5.9 mm (4.8–6.8 mm)	80% of corneal thickness (mean, 475 ± 59 μm)	9	Post-PKP	3 months	9.80 ± 1.90	5.20 ± 1.50	46.90%
Cleary et al. (2013) [11]	Penetrating	✓	7.8–8 mm	65%–75%	6	Post-PKP	4.5 months	9.8 ± 2.9	4.5 ± 3.2	54.10%
Trivizki et al. (2015) [68]	Penetrating	✓	5.79 ± 0.32 mm (5.5–6 mm)	80% of the minimal graft thickness	27	-	1 year	8.43 ± 2.8 (refractive)	3.31 ± 1.39 (refractive)	60.70%
Hoffart et al. (2009) [18]	Penetrating	✓	6.5 mm	75%	10	-	6 months	7.01 ± 3.02	3.97 ± 2.38	43.37%
Kymionis et al. (2009) [20]	Penetrating	✓	6.5 mm	75%	1	-	6 months	4.4	0.67	84.77%
Hashemian et al. (2017) [7]	Penetrating	✓	grafts diameter 7.25–8.25 mm incision 1 mm inside graft-host interface	85% of local corneal thickness	23	Post-PKP/DALK	6 months	7.75 ± 2.75	3.69 ± 3.54	52.40%
Al Sabaani et al. (2016) [8]	Penetrating	✓	0.5–0.7 mm inside graft–host junction	75–85%	52	Post-PKP	13.77 ± 4.17 months	6.73 ± 2.49	4.35 ± 3.83	35.40%
Nubile et al. (2009) [10]	Penetrating	✓	1 mm from graft edge	90%	12	Post-PKP	6 months	7.16 ± 3.07 (refractive)	2.23 ± 1.55 (refractive)	68.85%
Fadlallah et al. (2015) [9]	Penetrating	✗	5.8–7.5 mm (0.5 mm inside graft–host junction)	90% of corneal thickness (390–590 μm)	62	-	2 years	7.1 ± 1.72	3.5 ± 3.1	50.70%
Kumar et al. (2010) [6]	Penetrating	✗	1 mm less than graft diameter (0.5 mm within graft–host junction)	90%	37	Post-PKP	7.2 months	7.46 ± 2.7	4.77 ± 3.29	36.10%
Venter et al. (2013) [5]	Intrastromal	✗	7 mm	60 μm from the epithelium to 80% depth	47	Post-LASIK	7.6 ± 2.9 months	1.15 ± 0.43	0.49 ± 0.37	57.40%
Viswanathan and Kumar (2013) [12]	Intrastromal	✗	0.5 mm within the graft–host	60 μm below the epithelium 90% of stroma	2	Post-PKP	4 months	11.9 (OD)	4.1 (OD)	65.50%
								10.4 (OS)	1.12 (OS)	89.42%
Shalash et al. (2015) [36]	Intrastromal	✗	7.0 mm optical zone (0.5–1.0 mm inside graft–host interface)	95% of corneal thickness	20	Post-PKP	1 step	5.39 ± 0.98	2.46 ± 1.07	54.40%
							1 month		0.85 ± 0.87	84.20%
							1 year		1.05 ± 0.71	80.50%
Wetterstrand et al. (2015) [14]	Intrastromal	✗	6–7 mm	90 μm below epithelium and 10% intact posterior cornea	10	-	3 months	8.8 (5.1 to 16.0)	5.1 (1.1 to 8.5)	42.05%
				90 μm below epithelium and 125 μm intact posterior cornea	10	-		8.5 (3.9 to 10.5)	6.5 (4.4 to 9.9)	23.53%
Wetterstrand et al. (2013) [15]	Intrastromal	✗	6–7 mm	90 μm below the epithelium and 90% of stroma	16	Post-PKP	3 months	9.49 ± 4.78	4.41 ± 2.14	53.50%

*FSAK* = femtosecond laser-assisted astigmatic keratotomy; *PKP* = penetrating keratoplasty; *DALK* = deep anterior lamellar keratoplasty; *LASIK* = laser-assisted in situ keratomileusis

#### (iii) IFSAK

Intrastromal FSAK (IFSAK) is performed where the cut is within the stroma and does not reach Bowman’s layer. The absence of an open wound can avoid infection, wound gape, or epithelial ingrowth. Wetterstrand et al. [14] suggested that the desired intact posterior corneal margin should be close to 90 μm by balancing the measurement accuracy, protection of the endothelium, and efficacy. This allowed reduction of astigmatism up to 53% [15].

Among the studies of IFSAK, the changes in keratometric astigmatism have ranged from 0.66 D [5] to 9.28 D [12], with the percentages of astigmatic reduction ranging from 23.53% [14] to 89.42% [12]. The summary is presented in Table 1.

#### (iv) FSAK in eyes after Descemet stripping automated endothelial keratoplasty

Yoo et al. [16] reported a case treated with FSAK for post-Descemet stripping automated endothelial keratoplasty in which there was an approximate overcorrection of 7.5 D and refractive astigmatism changed from + 5.25 × 165 preoperatively to + 7.50 × 80 postoperatively. The authors commented that this massive correction of about 12.75 D was due to a full-thickness arcuate incision in the recipient cornea, as the 90% depth was calculated based on the total corneal thickness (i.e., recipient cornea + donor cornea). The authors recommended that the thickness of the donor graft must be excluded to avoid a full-thickness incision of the recipient cornea.

### Wedge resection for high astigmatism

Astigmatism after PKP usually ranges between 3 to 5 D [22], but some can have up to 20 D of astigmatism [23]. The wedge resection is a technique performed to correct high astigmatism, i.e., usually more than 10 D, which is much higher than that fixed through relaxing incisions, but the visual rehabilitation is longer. With wedge resection, the cornea is steepened rather than flattened. The surgery is performed by making two intersecting arcuate cuts based on two different arc lengths with varying angles of cut that intersect each other; a wedge of corneal tissue is excised from the flatter meridian to steepen the cornea. The width of the excision varies from 0.2 to 1 mm based on the amount of preoperative astigmatism; generally, every 0.05 mm of tissue removed corrects 1 D of astigmatism. Removal of defined widths and depths of tissue is difficult with manual methods. However, the femtosecond laser has facilitated such procedure with higher accuracy [24]. Besides, a trend toward myopic shift is observed due to a coupling effect. Suture tightness and removal are essential factors in accuracy or astigmatism correction with wedge resections.

### Stability in post-keratoplasty eyes

Fadlallah et al. [9] reported regression from 1 to 2 years postoperatively in their long-term study. The SIA changed from 3.28 D at 6 months to 3.5 D at 1 year to 2.86 D at 2 years postoperatively.

### Summary of status of post-keratoplasty eyes

Review of published articles (Table 1) reporting the results of FSAK performed after PKP/DALK revealed no significant differences in astigmatism reduction between procedures with opened penetrating wounds and those with closed penetrating wounds.

The general belief is that IFSAK has less of an effect than penetrating FSAK. Although different studies had different incisional depths, incisional arc lengths, and optical zone diameters, there is insufficient evidence to prove that penetrating correction produces a more significant effect than intrastromal correction. However, due to the limited number of studies and data that compared intrastromal AK with penetrating AK, more extensive studies with higher number of patients and longer follow-ups are required to prove this.

The advantages of performing an intrastromal procedure are almost no risk of infection, epithelial ingrowth, or wound gape. However, after PKP or DALK there is already an open wound, and, therefore, this advantage is less than in native eyes.

## FSAK in native eyes

### Efficacy

The amount of astigmatic correction generally is limited to 0.5 D to 1.5 D in native eyes, and most of the cuts are performed at an optical zone of 7.5 mm or more to prevent dysphotopsia. A summary is shown in Table 2.

**Table 2:** Reviewed articles of FSAK in native eyes

Reference	Type of FSAK	Open wound	Optical zone diameter	Incisional depth	Eyes (no.)	Follow-up	Mean keratometric astigmatism (preoperative) (D)	Mean keratometric astigmatism (postoperative) (D)	% Reduction
Chan et al. (2015) [27]	Penetrating	✗	8 mm	450 μm	54	2 months	1.33 ± 0.57	0.87 ± 0.56	34.60%
Chan et al. (2016) [33]	Penetrating	✗	8 mm	450 μm	50	2 months	1.35 ± 0.48	0.67 ± 0.54	50.40%
						2 years		0.74 ± 0.53	45.20%
Yoo et al. (2015) [25]	Penetrating	✗	9 mm	85%	23	1 month	1.315 ± 0.131	0.963 ± 0.136	26.80%
						5 months		0.874 ± 0.135	33.50%
Loffler et al. (2017) [44]	Penetrating	✓	9 mm	80%	27	3 months	0.96 ± 0.26	0.53 ± 0.35	44.80%
Wang et al. (2016) [28]	Penetrating	✓	8 mm	90%	51	1 month (35 eyes)	1.41 ± 0.42	-	46.10%
						3 months (28 eyes)		-	47.50%
Abbey et al. (2009) [26]	Penetrating	✓	6.75 mm	400 μm	2	1 year	5.92 (OD)	3.6 (OD)	39.20%
							5.8 (OS)	2.4 (OS)	58.62%
Venter et al. (2013) [5]	Intrastromal	✗	7 mm	60 μm from epithelium to 80% depth	65	7.6 ± 2.9 months	1.22 ± 0.49	0.58 ± 0.41	52.50%
Day et al. (2016) [29]	Intrastromal	✗	8 mm	20%–80%	319	1 month	1.24 ± 0.44	0.79 ± 0.41	36.30%
Day et al. (2016) [30]	Intrastromal	✗	8 mm	20%–80%	196	1 month	1.21 ± 0.42	0.74 ± 0.38	38.80%
Rückl et al. (2013) [17]	Intrastromal	✗	7.5 mm	100 μm from epithelium and endothelium	15	6 months	1.50 ± 0.47	0.63 ± 0.34	58%

*FSAK* = femtosecond laser-assisted astigmatic keratotomy

#### (i) Penetrating FSAK

As shown in Table 2, the keratometric astigmatic changes ranged from 0.352 D [25] to 3.4 D [26], and the percentages of the astigmatic reductions ranged from 26.8% [25] to 58.62% [26]. Chan et al. [27] performed penetrating FSAK (wound not opened) in 54 eyes that underwent cataract surgery. The authors set the laser arc length according to the corneal astigmatic magnitude to be corrected, based on their nomogram modified from the Wallace limbal relaxing incision (LRI) nomogram. The authors concluded that there was a trend toward undercorrection when target-induced astigmatism (TIA) was 1 D or more and overcorrection when it was less than 1 D. This implied that the nomogram might need further adjustment. Moreover, Wang et al. [28] reported that older age, longer incisional length, and horizontal incisions in eyes with preoperative against-the-rule (ATR) corneal astigmatism predicted a greater postoperative astigmatic correction.

#### (ii) IFSAK

Among the IFSAK studies reviewed in this article, the keratometric astigmatic changes ranged from 0.45 D [29] to 0.87 D [17], and the percentages of astigmatic reduction ranged from 36.3% [29] to 58% [17].

Day et al. [30] performed IFSAK in 196 eyes. The nomogram for the laser arc length was based on the degree of preoperative corneal astigmatism, age, and type of astigmatism. The corneal astigmatism decreased by 39% from 1.21 D preoperatively to 0.74 D postoperatively. Vector analysis showed under-correction of astigmatism (mean correction index, 0.63 [< 1]; mean magnitude of error, − 0.47 [< 0]). The angle of error was small, i.e., 3 degrees. The study did not reveal significant risk factors for astigmatic under- or overcorrection, which implied that the nomogram might include other factors in the future to improve the accuracy.

Day and Stevens [31] performed IFSAK in 87 eyes during cataract surgery and compared the results to a group of eyes undergoing cataract surgery without IFSAK in 176 eyes. A personal nomogram for the laser arc length was used. At 1 and 6 months postoperatively, the IFSAK group had significantly higher SIA than the non-IFSAK group (0.78 D vs. 0.43 D, respectively, at 1 month; 0.69 D vs. 0.32 D at 6 months), which indicated that IFSAK reduced the corneal astigmatism during cataract surgery. The regression effect was comparable between the groups.

Rückl et al. [17] performed IFSAK in 16 eyes without cataract surgery, with a TIA of 1.59 D. At 6 months postoperatively, corneal astigmatism decreased by 58% from 1.50 D to 0.63 D. Vector analysis showed a mean SIA of 1.59 D and correction index of 1.0. However, it is worth noting that two (13%) eyes had strong overcorrection (correction index close to 2.0) and four (25%) eyes had extensive under-correction (correction index close to 0.5), that is, six (37%) of 16 eyes had an undesirable correction. However, the authors did not report the individual preoperative data from these eyes that might help identify the risk factors for inaccurate correction. The corneal astigmatism was stable throughout the study period postoperatively at 1 day, 1 week, and 1, 3, and 6 months.

### Stability in native eyes

Placement of manual LRIs has been shown to be stable for up to 3 years [32].

#### (i) Penetrating FSAK

Chan et al. [33] performed penetrating AK (wound not opened) in 50 eyes. The mean preoperative TIA was 1.35 ± 0.48 D, which decreased to 0.67 ± 0.54 D at 2 months and 0.74 ± 0.53 D at 2 years postoperatively. There was no significant difference between the postoperative corneal astigmatism over 2 years and no difference in the magnitude of error, absolute angle of error, and higher order aberrations postoperatively to 2 years.

#### (ii) IFSAK

Rückl et al. [17] reported stable corneal astigmatism with IFSAK from 1 day (0.61 ± 0.43 D) to 6 months (0.33 ± 0.42 D) postoperatively.

Day and Steven [31] compared the SIA resulting from IFSAK during cataract surgery and standard femtosecond laser-assisted cataract surgery to exclude astigmatism induced by the main incision and side ports in cataract surgery. Regression analysis at 1 and 6 months postoperatively showed small but significant regression with standard cataract surgery (0.11 D) and cataract surgery with IFSAK (0.09 D); however, the values were low and of little clinical relevance.

### Summary of status of native eyes

The differences in astigmatic reduction are not significant among penetrating wound open, penetrating wound closed, and intrastromal correction for native eyes (Table 2). Larger randomized controlled trials of IFSAK with more extended follow-up periods are needed.

## FSAK in post-trabeculectomy eyes

Kankariya et al. [34] reported a case of mixed astigmatism induced after trabeculectomy treated with FSAK. A penetrating paired incision (wound open) was made at the 7.0-mm optical zone. Corneal astigmatism decreased from 4.15 D to 0.81 D, and the UCVA improved from 20/200 to 20/60, which was the same UCVA as before trabeculectomy. The intraocular pressure was maintained, and the trabeculectomy bleb morphology was preserved.

## Efficacy of combined intrastromal AK and laser-assisted in situ Keratomileusis

Loriaut et al. [35] and Shalash et al. [36] reported another technique to correct native eyes, or those that underwent PKP with high astigmatism by performing IFSAK after the creation of a laser-assisted in situ keratomileusis (LASIK) flap followed 1 to 3 months later by excimer laser photoablation. While this technique permits correction of a broader range of high astigmatism and can reduce the astigmatism by over 80%, epithelial ingrowth and microperforations are considerations.

## Improving efficacy and safety

### Nomogram

The commonly used MAK nomograms are the Lindstrom nomogram [37] and the Hanna nomogram [38] for correcting astigmatism after PKP. The zone diameter, incisional depth, arc length, and age are the variables that determine the incision. More central placement of the incision, greater depth, longer incision, and older age have resulted in a higher effect of the astigmatic correction.

A coupling effect [39] must be considered when planning astigmatism surgery that predicts the impact of astigmatic incisions on the spherical equivalent refraction (SE). The coupling ratio is defined as the ratio of the amount of flattening of the incised meridian to the amount of steepening of the opposite meridian. Flattening is created at the meridian of the incision while steepening is induced at the meridian 90 degrees away. If the coupling ratio is 1, the SE will not change. When the coupling ratio is greater than 1 and less than 1, the results are, respectively, a hyperopic shift and a myopic shift. Incisional arc lengths of 30 to 90 degrees result in a coupling ratio of close to 1; arc lengths less than 20 degrees have a coupling ratio greater than 1, whereas those greater than 100 degrees have a coupling ratio less than 1 [2].

#### (i) Nomogram of FSAK in post-PKP eyes

Based on published data, the most frequently used nomogram for FSAK after PKP is the topographic map method [6, 7, 9, 10]. In this nomogram, the lengths of the relaxing arcuate incisions are ascertained by the borders of the steep semi-meridians, and the incisions are placed either 0.5 mm [6, 9] or 1 mm [7, 10] within the graft-host junction. The other commonly used nomogram is the Hanna nomogram with or without modification [4, 13, 18], which was designed originally for manual mechanical AK [40]. The accuracy and predictability varied considerably in eyes after PKP; hence, surgeons often have to make adjustments based on experience and surgical technique. Few reports have been published on the appropriate nomograms to use in eyes after PKP or in native eyes.

Another nomogram developed by St. Clair et al. [13] was tested on 89 eyes, which is currently the most significant sample reported in similar studies. According to the nomogram, the incisional depth, arc length, and optical zone diameter changed concerning the difference between the steepest and flattest K values. The mean refractive cylinder decreased significantly from 6.77 ± 2.80 D to 2.85 ± 2.57 D. A trend of under-correction of 3.62 D was reported with a low incidence of overcorrection, 6.7%, which was comparable to the 8% to 10% reported [6, 41]. A coefficient of determination of the generated nomogram was 0.67, that is, 67% of the variation in accuracy can be explained by preoperative astigmatism and incisional parameters, and the other 33% is recognized as unknown variables or inherent variability.

St. Clair et al. [13] postulated that the effect of AK on astigmatism after PKP differs from that on native corneas because of the oblique and irregular tension in the corneal graft, resulting in a less-than-perfect tissue distribution during PKP. The age of the donor graft also might affect the result, since older corneas are stiffer than younger donor corneas.

Another nomogram of beveled FSAK developed by Cleary et al. [11] used a side-cut angle of 135 degrees instead of 90 degrees. The authors hypothesized that a beveled incision allows the anterior cornea to slide forward, thus reducing astigmatism and preventing wound gape. Despite the small sample size of six eyes, it provides a good starting point for surgeons who want to attempt beveled FSAK.

The accuracy of these nomograms that are explicitly designed for use during FSAK after PKP is not yet ascertained. Large-scale randomized studies are needed to provide evidence to support or refine these nomograms.

#### (ii) Nomogram of FSAK in native eyes

Abbey et al. [26] reported a case of native eyes treated with penetrating FSAK based on their modified version of the Lindstrom nomogram. The manifest astigmatism decreased from − 3.50/+ 5.25 × 89 preoperatively to − 1.75/+ 2.75 × 90 postoperatively in the right eye and from − 3.50/+ 5.25 × 83 to − 1.75/+ 2.25 × 85 in the left eye. Topography showed improved astigmatism with and unchanged axis. Its efficacy, however, had not been evaluated.

### Consideration of the posterior cornea

In native eyes, ATR astigmatism was present in 86.6% of the posterior cornea [42]. Thus, overcorrection of the ATR astigmatism and under-correction of with-the-rule (WTR) astigmatism by 0.75 D during cataract surgery was suggested. Mild residual WTR astigmatism is preferred over ATR, as it allows better distance and near vision [43].

Löffler et al. [44] analyzed the effect on the anterior, posterior, and total corneal astigmatism in eyes that underwent penetrating FSAK and found a significant reduction in astigmatism in the anterior and total corneal astigmatism but not in the posterior corneal astigmatism. These results are consistent with the finding that the contribution of the posterior cornea was significantly lower (0.26 ± 0.10 D) compared to the anterior (0.97 ± 0.30 D) and total corneal (0.96 ± 0.26 D) astigmatism. While the posterior cornea does not affect the “corneal” astigmatic correction with FSAK, the effect of the posterior cornea on the total “refractive” astigmatism should be considered when performing cataract refractive surgery and FSAK simultaneously. However, when performing FSAK on patients who underwent previous cataract surgery, the refractive result is purely on the anterior cornea.

Wang et al. [28] reported 14.9% overcorrection 1 month after penetrating FSAK (wound open) in native eyes; two-thirds of these overcorrected eyes had WTR corneal astigmatism preoperatively. The authors assumed that these overcorrections resulted from not considering the posterior cornea. A new nomogram was developed to account for the effect of the posterior cornea [28], which reduced the overcorrection to 6.7%; however, further validation of the nomogram is needed.

Recently, Day et al. [30] reported the results of IFSAK based on a personal nomogram that considered the posterior cornea. The arc length was increased by 5 degrees for ATR astigmatism but decreased by 5 degrees for WTR astigmatism, which resulted in a higher corrective index of astigmatism of 63% and lower overcorrection of 7%.

### Cyclotorsion

Another factor that can affect astigmatic correction is the accurate placement of the astigmatism axis; every degree of cyclotorsion error can cause under-correction of 3.3% [45]. Modern femto-cataract lasers already can match the astigmatism axis to the iris registration preoperatively and then align the FSAK to the iris pattern to achieve better accuracy [46].

### Corneal biomechanics

Aside from the effects of zone diameter, arc length, incisional depth, and age on the incisions in traditional and modified nomograms, the impact of other corneal parameters on the incisions has been studied.

Day and Stevens [29] studied the preoperative parameters of 319 eyes undergoing cataract surgery with intrastromal AK to identify the factors predictive of the accuracy of FSAK. The corneal biomechanics evaluated included corneal hysteresis (CH), which reflects the corneal damping ability, and corneal resistance factor (CRF), which indicates the overall corneal rigidity. Multivariable regression analysis of the SIA showed that CH and CRF were independent predictors of SIA, such that the average SIA decreased by 0.06 D for every further diopter increase of CH and increased by 0.04 D for every additional diopter increase of CRF. Also, the WTR astigmatism had an average SIA 0.13 D more than ATR astigmatism. Although the study had a short follow-up period of 1 month postoperatively, a previous study found minimal regression associated with FSAK [17, 31]. Therefore, the findings indicated that the corneal biomechanical parameters, CH and CRF, might be included in later nomograms to improve accuracy.

Furthermore, it was not recommended to place the incisions in the recipient corneas because the corneal biomechanics might be altered as a result of scarring at the graft-host junction. The effect of relaxing incisions in the recipient cornea was supposed to be blocked by the new limbus formed by the keratoplasty wound [47].

### Anterior-segment OCT

Anterior-segment OCT (AS-OCT) is useful for both preoperative planning and postoperative monitoring of FSAK patients. In FSAK, one parameter that offsets the amount of astigmatic correction is the incisional depth, i.e., the deeper the incision, the more significant the effect. For penetrating incisions, if the cuts are more anterior than projected, there might not be sufficient depth to attain the desired astigmatic correction [48]. Anterior displacement of the intrastromal incision can lead to a higher risk of anterior perforation, significant overcorrection, irregular astigmatism, and visual loss [48]. Detailed AS-OCT measurement of the peripheral corneal thickness enables precise surgical planning of the incisional depth, which prevents full-thickness corneal perforation. Ideally, dynamic AS-OCT would be even more beneficial by allowing real-time measurement and adjustment of the incision. AS-OCT assessment of the incisional depth 3 weeks postoperatively might be helpful.

It facilitates comparison and monitoring of any mismatch between the programmed and achieved incisional depths [19, 26]. Furthermore, structural changes in the corneal wound can be studied to rule out any effects from wound healing.

## Safety

### Complications in post-keratoplasty eyes

#### (i) Overcorrection

The rates of overcorrection in patients who underwent FSAK after PKP have been reported to be 19.4% [9], 23% [8], and 43.5% [7]. Overcorrection after PKP can be managed by tightening the sutures; however, the effect is unreliable.

Interestingly, in earlier studies [10, 18, 21, 49] in which shorter arc lengths were used (up to 80 degrees), no overcorrection was reported. The recent aggressive approach to maximize the amount of correction appears unpredictable. Possible long-term (5 to 10 years) undesirable effects of this extensive weakening of the donor graft after FSAK remain unknown. The ultimate goal of AK is to reduce astigmatism to a level that visual aids are acceptable to patients. Therefore, a balance between residual astigmatism and risk of visual acuity loss/complications should be evaluated in each patient.

#### (ii) Visual loss

Loss of two or more lines of the BCVA was reported in eyes after PKP when penetrating FSAK was performed, ranging from 3.2% to 20% [9, 13, 44]. No visual loss has been reported in association with IFSAK.

#### (iii) Posterior perforation

The incidence rates of microperforations in eyes after PKP undergoing penetrating FSAK have been reported to be 3.2% to 8.7% [7–9]. The microperforations were self-sealing, and the anterior chambers were maintained with no postoperative sequelae. In most cases, application of a bandage contact lens was adequate. Al Sabaani et al. [8] reported that only one (1.9%) case required resuturing of the AK wound.

A higher prevalence of microperforations (35%) was reported in eyes that underwent IFSAK with the creation of a LASIK flap [36]. The intrastromal AK incision was made at a depth of 95% of the local corneal thickness (guided by intraoperative pachymetry) after the flap was created and lifted. There were no intraoperative leaks, and a contact lens was applied by the end of surgery with no postoperative sequelae.

Hashemian et al. [7] proposed that the microperforations could have resulted from mechanical stress induced by a Sinskey hook used to separate the tissue bridges within the margins of the cut rather than from the primary full-thickness femtosecond laser cut. This literature review did not identify any reports of macroperforations. If a full-thickness perforation occurs, the wound should not be opened and allowed to heal; AK should be performed again later at another optical zone.

#### (iv) Infectious keratitis

Infections are more likely to develop in eyes that underwent PKP because the eyes are more immunocompromised [50]. The infection rates associated with FSAK after PKP have ranged from 0% to 4.8% [8, 9, 13]. The infections typically were observed between 6 months and 1 year postoperatively, and all resolved with topical antibiotic therapy.

Occasionally, fibrosis does not develop (even over the long term) and if the epithelium is compromised infection can occur as late as 15 years later [51]. We are unaware of any infectious keratitis associated with IFSAK as there is no open wound. It has been suggested that closed wounds minimized the infection risk [10, 33] and postoperative discomfort [33].

#### (v) Endophthalmitis

Only one case of endophthalmitis was reported after FSAK after PKP [9] with no previous clinical evidence of wound leakage. Endophthalmitis developed 5 days after FSAK, and the patient was treated with 9 D of cylinder. The endophthalmitis resolved with intravitreal antibiotic therapy but the patient lost two lines of BCVA.

#### (vi) Allograft rejection

St. Clair et al. [13] reported a 2.2% incidence of graft rejection in eyes that underwent penetrating FSAK. Fadlallah et al. [9] reported a 4.8% (3/62 eyes) incidence of graft rejections that occurred 3 months to 1 year postoperatively; all resolved after treatment with topical antibiotic steroids with no postoperative sequelae.

### Complications in native eyes

#### (i) Overcorrection

Wang et al. [28] reported an incidence rate of overcorrection of 14.9% at 3 months postoperatively. Two-thirds of the 14.9% overcorrected eyes had WTR corneal astigmatism preoperatively, and the authors presumed that the overcorrection might have resulted from ignoring the effect of the posterior corneal astigmatism.

#### (ii) Anterior gas breakthrough

Most small amounts of the anterior gas breakthrough do not cause problems. However, Kankariya et al. [52] reported a case of anterior gas breakthrough during IFSAK, in which irregular astigmatism was induced. There was also a significant overcorrection of corneal astigmatism from 0.84 × 176 preoperatively to 4.97 × 70 1 month postoperatively and a decrease in the BCVA from 20/20 to 20/30.

#### (iii) Visual loss

Only one report of visual loss in FSAK performed on native eyes from 20/20 to 20/30 was reported as mentioned previously [52].

#### (iv) Suction loss

An intraoperative suction loss might affect the accuracy of the incision. Rückl et al. [17] reported a case of suction loss due to movement of the patient’s head. The incisional alignment was affected but remained purely intrastromal, with no subsequent visual loss.

#### (v) Misaligned position of incisions

During FSAK, since the femtosecond laser system identifies the ocular structure on OCT scans, good-quality OCT scans and ocular stability during the laser firing stage are vital to ensure the correct position of the incision. During manual AK, surgeons can cut through the visual axis if the patient inadvertently moves during the surgery, causing visual loss. Such scenario is unlikely in FSAK since most machines stop quickly when suction is lost [53].

#### (vi) Endothelial cell loss

There is concern that femtosecond laser energy close to the endothelium can affect the survival of the endothelial cells. However, Rückl et al. [17] and Hoffart et al. [41] reported no significant endothelial cell loss after FSAK.

#### (vii) Ectasia

Wellish et al. [54] reported a case of corneal ectasia after multiple manual keratotomy procedures. Twelve enhancement procedures were performed to treat residual astigmatism after myopic astigmatism treated with manual AK, which resulted in a double hexagonal keratotomy. A conically shaped protrusion of the central cornea, Munson’s sign, diffuse subepithelial scarring, and central corneal thinning were seen. Therefore, repeated AK for enhancement should be performed cautiously. Ectasia has not been reported after FSAK.

### Other surgical treatment options for astigmatism after PKP

Other refractive surgeries including LASIK [55–59], laser subepithelial keratomileusis/photorefractive keratectomy [57, 59–61], toric intraocular lenses (IOLs) [62, 63], and intrastromal corneal ring segments [64–66] are sometimes used to correct astigmatism after PKP.

Until now, only one report compared penetrating FSAK and toric IOLs. In that study, Yoo et al. [25] studied the clinical efficacy and safety of FSAK (9-mm optical zone, 85% depth, closed wound) performed after cataract surgery and compared them with toric IOL implantation in cataract patients with corneal astigmatism. The authors found no significant difference in the residual refractive astigmatism between the two treatment methods. These results indicated that either manual or femtosecond laser AK could be substituted for toric IOL implantation in patients with mild corneal astigmatism.

## Conclusion

FSAK reduces astigmatism in post-keratoplasty eyes with high regular or irregular astigmatism. Complications are rare and manageable. The predictability varies, and improvement of the BCVA is not guaranteed [8]. VA losses have been reported. Large-scale, randomized studies using newly developed nomograms with long-term follow-up are needed.

For native eyes undergoing femtosecond laser-assisted cataract surgery, IFSAK should be the choice for astigmatic correction, and until better nomograms become available, IFSAK should be reserved to treat low amounts of astigmatism (< 1.5 D).

For patients who have already had their cataracts removed or those who underwent PKP, the effect of astigmatic correction is almost completely on the anterior cornea, and the posterior cornea contributes very little. However, when performing refractive astigmatic correction, i.e., FSAK with cataract surgery, the effect of the posterior cornea on astigmatism should be considered.

Patients should be instructed to avoid rubbing their eyes to prevent sight-threatening complications. Notably, in the 6 months postoperative period, the penetrating incisions can become infected, even when the wound is closed. It is best not to open the penetrating incision also though the effect might be greater since it can lead to late infections (up to 15 years). The patient should be informed of this risk preoperatively.

The effectiveness of IFSAK seems to be comparable to that of penetrating AK. Because of the superior safety profile of IFSAK, more attention should be paid to this corrective procedure.

## Definitions

As defined by vector analysis with the Alpins method [67],

- SIA: surgically induced astigmatism is defined as the amount of astigmatism the surgery actually induced.
- TIA: target induced astigmatism is defined as the amount of astigmatism the surgeon intended to induce, it is equal to preoperative measured corneal astigmatism if the target is to clear all astigmatism.
- DV: difference vector is defined as the amount of astigmatism that has to be postoperatively corrected to finally reach the intended target astigmatism, it is equal to the postoperative astigmatism.
- The coefficient of determination [13] is the proportion of the variance in the dependent variable that is predictable from the independent variable(s).

## Abbreviations

AK: Astigmatic keratotomy
AS-OCT: Anterior segment OCT
ATR: Against-the-rule
BCVA: Best-corrected visual acuity
CH: Corneal hysteresis
CRF: Corneal resistance factor
D: Diopters
DALK: Deep anterior lamellar keratoplasty
FSAK: Femtosecond laser-assisted astigmatic keratotomy
IFSAK: Intrastromal FSAK
IOL: Intraocular lens
LASEK: Laser-assisted subepithelial keratectomy
LASIK: Laser-assisted in situ keratomileusis
LRI: Limbal relaxing incisions
PKP: Penetrating keratoplasty
SE: Spherical equivalent refraction
SIA: Surgical induced astigmatism
TIA: Target induced astigmatism
UCVA: Uncorrected visual acuity
WTR: With-the-rule
